# The serum uric acid/high-density lipoprotein cholesterol ratio: a novel predictor for the presence of abdominal aortic aneurysm

**DOI:** 10.3389/fcvm.2024.1481872

**Published:** 2024-11-11

**Authors:** Wei Li, Songyuan Luo, Wenhui Lin, Xiaolu Hu, Dan Zhou, Wenmin Xu, Yingling Zhou, Jianfang Luo, Yingqing Feng

**Affiliations:** ^1^ Department of Cardiology, Guangdong Provincial People’s Hospital, Zhuhai Hospital (Jinwan Central Hospital of Zhuhai), Zhuhai, China; ^2^ Department of Cardiology, Guangdong Cardiovascular Institute, Guangdong Provincial Key Laboratory of Coronary Heart Disease Prevention, Guangdong Provincial People’s Hospital, Guangdong Academy of Medical Sciences, Guangzhou, China; ^3^ Department of Internal Medicine, Guangdong Provincial People’s Hospital, Zhuhai Hospital (Jinwan Central Hospital of Zhuhai), Zhuhai, China

**Keywords:** abdominal aortic aneurysm, serum uric acid, high-density lipoprotein cholesterol, predictor, screening

## Abstract

**Objective:**

Robust evidence has demonstrated that inflammation plays an important role in the occurrence and development of abdominal aortic aneurysms (AAA). The serum uric acid (UA)/high-density lipoprotein cholesterol (HDL-C) ratio (UHR) has recently been recognized as a new biomarker for evaluating inflammatory and anti-inflammatory interactions. However, whether UHR is associated with AAA remains unclear. This study aimed to explore the association between UHR and presence of AAA.

**Methods:**

We prospectively performed a hospital-based and community-based AAA screening program using ultrasonography in 9,064 individuals at Guangdong Provincial People’s Hospital and two communities in China. Logistic regression analysis was used to explore the association between UHR and presence of AAA. In addition, the restricted cubic spline (RCS) regression method was used to visually investigate the dose-response relationship between UHR and the presence of AAA. Propensity score matching (PSM) analysis was conducted to adjust for baseline variations and diminish selection bias, and subgroup analysis was performed to investigate the consistency of the conclusions.

**Results:**

The prevalence of AAA was 2.45% (222/9,064) in the present study. The optimal cut-off value of UHR was 17.0%, which was selected according to the receiver operator characteristic curve. The prevalence of AAA was 3.96% in the high-UHR group (UHR ≥ 17%) and 1.54% in the low-UHR group (UHR < 17%) (*P* < 0.001). After adjusting for other relevant clinical covariates, UHR was independently associated with the presence of AAA, either as a continuous variable (odds ratio [OR] 1.03, 95% confidence intervals [CI] 1.01–1.05, *P* < 0.001) or as a categorical variable (OR 1.63, 95% CI 1.18–2.26, *P* = 0.003). The RCS curve showed a nonlinear dose-response relationship between UHR and the presence of AAA. Moreover, the positive correlation between UHR and the presence of AAA remained significant after PSM and subgroup analyses.

**Conclusions:**

UHR was positively associated with the presence of AAA, and there was a non-linear dose-response relationship between them. Thus, UHR may serve as a novel and reliable predictor of AAA.

## Introduction

Abdominal aortic aneurysm (AAA) is an important cause of preventable death in the elderly, with 80% overall mortality in the event of rupture (1). Four large-scale randomized controlled trials, with AAA prevalence rates of 4%–7.2%, indicated that screening for AAA in elderly men would reduce AAA-specific mortality by 40% after 3–5 years of follow-up (2–6). Considering the balance between cost-effectiveness and incidence, many guidelines recommend the use of ultrasonography for AAA screening in high-risk populations (7–9). However, epidemiological studies have demonstrated that Asians have a lower prevalence of AAA than that reported in Caucasians (10–12). Several AAA screening programs in China have indicated that the prevalence of AAA is only 0.11%–0.90% (10, 13). As the cost-effectiveness of screening for AAA is affected by disease prevalence, future screening strategies for AAA in Chinese individuals should target high-risk individuals. Therefore, exploring predictors for high-risk populations will help improve the cost-effectiveness of AAA screening.

The serum uric acid (UA)/high-density lipoprotein cholesterol (HDL-C) ratio (UHR) has recently been recognized as a new biomarker for evaluating inflammatory and anti-inflammatory interactions. Several studies have reported a relationship between UHR and diabetes as well as cardiovascular mortality in patients on peritoneal dialysis (14, 15). Genetic and epidemiological studies have indicated that elevated serum UA levels play an important role in the development and progression of atherosclerotic cardiovascular disease (ASCVD), through inflammatory responses, oxidative stress and endothelial dysfunction (16–18). HDL-C is a plasma lipoprotein that has anti-inflammatory and antioxidant properties. Endothelial protection and reverse cholesterol transport form the core function of HDL-C against atherosclerosis (19). Emerging evidence has demonstrated that UA and HDL-C have opposite effects on atherosclerosis, and both high UA and low HDL-C levels are associated with increased ASCVD risk and mortality (20, 21). Atherosclerosis is the most common cause of cardiovascular diseases, such as coronary artery disease (CAD), stroke, peripheral arterial disease (PAD), and AAA. Robust evidence has shown that inflammatory mechanisms play important roles in the occurrence and development of AAA (22). However, whether UHR is associated with AAA remains unclear.

Therefore, we explored the association between UHR and AAA using an AAA screening program in China.

## Methods

### Study population

**Figure 1:**
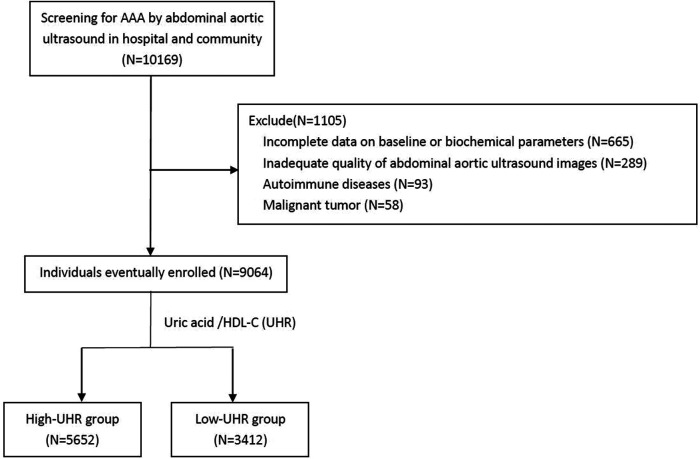
Flow chart demonstrating the inclusion of individuals in the study. AAA, abdominal aortic aneurysm; UHR, uric acid to high-density lipoprotein cholesterol ratio; HDL-C, high-density lipoprotein cholesterol.

An AAA screening program was conducted from June 2019 to June 2021 in the Department of Cardiology of Guangdong Provincial People's Hospital, Guangzhou, China, and in two communities from Dongguan, China. The study was approved by the Ethics Committee of Guangdong Provincial People's Hospital, and written informed consent was obtained from all individuals in the study. AAA screening using abdominal aortic ultrasound was performed in 10,169 individuals aged >45 years. The exclusion criteria were as follows: (i) incomplete data on baseline or biochemical parameters, (ii) inadequate quality abdominal aortic ultrasound images, and (iii) self-reported history of autoimmune diseases or malignant tumors. In total, 9,064 individuals (4,800 from the hospital and 4,264 from the community) were enrolled in the final analysis (Figure 1).

### Demographic data and history

Demographic data and history were recorded using standardized questionnaires that included sex, age, self-reported smoking and drinking history, hypertension, diabetes, CAD, stroke, and family history (first-degree relatives diagnosed with AAA). Hypertension was defined as systolic blood pressure (SBP) ≥140 mmHg and/or diastolic blood pressure (DBP) ≥90 mmHg or if the patient had a history of hypertension or current use of antihypertensive medications. Diabetes was defined as fasting plasma glucose (FPG) ≥7.0 mmol/L or random plasma glucose ≥11.1 mmol/L or hemoglobin A1c (HbA1C) ≥6.5%, or use of hypoglycemic drugs. CAD was defined by coronary angiography or coronary computed tomography angiography as the presence of stenosis ≥50% of the lumen diameter of at least one major coronary vessel, or if the patient had a history of CAD.

### Biochemical measurements

Venous blood was collected after overnight fasting and analyzed with standard laboratory methods by trained medical personnel, measuring the following laboratory parameters: white blood cells (WBC), platelets (PLT), hemoglobin (HB), fasting plasma glucose (FPG), blood urea nitrogen (BUN), creatinine (Cr), alanine aminotransferase (ALT), aspartate aminotransferase (AST), UA, triglyceride (TG), total cholesterol (TC), low-density lipoproteins cholesterol (LDL-C) and HDL-C. UHR was calculated as [UA (mg/dl)/HDL-C (mg/dl)] × 100%. eGFR was computed using the Modification of Diet in Renal Disease (MDRD) formula.

### Ultrasound screening of the abdominal aorta

Ultrasound screening of the abdominal aorta was performed as we previously described (23). All studies were reviewed by two experienced echocardiographers who were blinded to patient demographics. A third echocardiographer was advised in cases of disagreement, and the majority view was adopted. According to the latest guidelines (1, 24), the maximum infrarenal anteroposterior aortic diameter was measured from the outer edge to the outer edge. The AAA diagnosis was based on a maximum abdominal aortic diameter measurement of >30 mm.

### Statistical analysis

Continuous variables are presented as mean ± standard deviation or median (quartiles 1 through 3) according to the distribution characteristics and compared using the Student's *t*-test or Mann-Whitney test. For categorical variables, data were expressed as percentages and analyzed using the chi-squared test or Fisher's exact test.

A logistic regression model was used to show the results of different adjusted models to evaluate the association between UHR and the presence of AAA using odds ratios (OR) and 95% confidence intervals (95% CI). In this step, we performed three logistic regression models: model 1 was unadjusted, model 2 was adjusted for age and sex, and model 3 was adjusted for age, sex, smoking, hypertension, diabetes, CAD, stroke, eGFR, WBC, PLT, HB, ALT, AST, FPG, TC, TG, and LDL-C. First, the UHR was entered as a continuous variable. Second, UHR was modeled as a categorical variable with the optimal cutoff evaluated using the operating characteristic curve (ROC). The UHR at the maximum Youden Index was selected as the cut-off value. Moreover, we categorized the patients into four groups according to the quartiles of UHR to enhance clinical utility and repeated the analysis.

In addition, a propensity score matching (PSM) analysis was conducted using 1:1 nearest-neighbor matching with a caliper of 0.01 to adjust for baseline variations and diminish selection bias between the low- and high-UHR groups. The propensity score was calculated for each patient based on a logistic regression model, using the variables listed in Table 1 (including age, sex, smoking, hypertension, diabetes, CAD, stroke, eGFR, WBC, PLT, HB, ALT, AST, FPG, TG and LDL-C). The standardized mean difference (SMD) was used to measure the difference between groups after PSM. A maximum SMD of 0.10, or even 0.15, is usually regarded as appropriate.

**Table 1:** Baseline characteristics stratified by serum uric acid to high density lipoprotein cholesterol ratio (UHR) before and after propensity score matching.

Variables	Unmatched groups				Propensity score matched groups			
	UHR < 17% (*N* = 5,652)	UHR ≥ 17% (*N* = 3,412)	SMD	*P*	UHR < 17% (*N* = 2,452)	UHR ≥ 17% (*N* = 2,452)	SMD	*P*
Prevalence of AAA	87 (1.54%)	135 (3.96%)	0.15	**<0** **.** **001**	54 (2.20%)	106 (4.32%)	0.12	**<0**.**001**
Max.AAD (mm)	19.05 ± 4.05	20.01 ± 5.63	0.20	**<0**.**001**	19.45 ± 4.55	20.09 ± 5.88	0.12	**<0**.**001**
Age (years)	70.22 ± 6.94	70.70 ± 7.34	0.07	**0**.**002**	70.71 ± 7.37	71.03 ± 7.12	0.04	0.127
Male	3,217 (56.92%)	2,610 (76.49%)	0.42	**<0**.**001**	1,831 (74.67%)	1,792 (73.08%)	0.04	0.205
Smoker	1,194 (21.13%)	981 (28.75%)	0.18	**<0**.**001**	681 (27.77%)	654 (26.67%)	0.02	0.386
Hypertension	2,817 (49.84%)	2,063 (60.46%)	0.21	**<0**.**001**	1,436 (58.56%)	1,421 (57.95%)	0.01	0.664
Diabetes	1,010 (17.87%)	917 (26.88%)	0.22	**<0**.**001**	587 (23.94%)	583 (23.78%)	0.00	0.893
CAD	1,809 (32.01%)	1,571 (46.04%)	0.29	**<0**.**001**	1,045 (42.62%)	1,052 (42.90%)	0.02	0.368
Stroke	256 (4.53%)	219 (6.42%)	0.08	**<0**.**001**	143 (5.83%)	162 (6.61%)	0.03	0.261
WBC (10^9^/L）	6.87 ± 2.06	7.53 ± 2.36	0.30	**<0**.**001**	7.35 ± 2.27	7.30 ± 2.12	0.02	0.472
Platelets (×10^9^/L)	229.00 (193.00–272.00)	228.00 (188.00–272.00)	0.00	0.166	228.00 (190.00–271.02)	225.00 (184.00–267.00)	0.05	0.089
Hemoglobin (g/L)	134.06 ± 15.82	132.10 ± 19.95	0.11	**<0**.**001**	133.74 ± 17.29	133.93 ± 19.22	0.04	0.654
eGFR (ml/min × 1.73 m^2^)	84.81 (72.28–98.80)	71.34 (55.00–86.22)	0.65	**<0**.**001**	75.68 (64.18–87.42)	75.33 (60.29–89.42)	0.02	0.362
FPG (mmol/L)	5.87 ± 2.30	6.25 ± 2.69	0.15	**<0**.**001**	6.12 ± 2.60	6.25 ± 2.70	0.05	0.103
ALT (U/L)	19.00 (14.10–26.60)	20.60 (15.00–29.75)	0.07	**<0**.**001**	25.95 ± 24.45	25.58 ± 25.55	0.01	0.607
AST (U/L)	22.00 (18.30–27.00)	22.00 (18.00–28.50)	0.05	**0**.**009**	22.50 (18.80–28.00)	22.00 (18.00–29.00)	0.02	0.514
UA (umol/L)	391.00 (338.92–447.30)	476.00 (415.95–549.02)	1.44	**<0**.**001**	362.00 (309.00–415.00)	467.15 (409.98–537.82)	1.22	**<0**.**001**
TC (mmol/L)	4.90 ± 1.17	4.43 ± 1.20	0.40	**0**.**001**	4.76 ± 1.17	4.34 ± 1.17	0.35	**<0**.**001**
TG (mmol/L)	1.42 ± 0.91	1.97 ± 1.45	0.45	**<0**.**001**	1.64 ± 1.09	1.62 ± 0.93	0.03	0.373
LDL-C (mmol/L)	3.04 ± 0.99	2.82 ± 0.96	0.23	**<0**.**001**	2.85 ± 0.94	2.85 ± 0.96	0.01	0.836
HDL-C (mmol/L)	1.35 ± 0.34	0.93 ± 0.20	1.51	**<0**.**001**	1.29 ± 0.31	0.94 ± 0.19	1.37	**<0**.**001**

Values are given as number (percentage), mean ± SD or median (quartiles 1–3). AAA, abdominal aortic aneurysm; ALT, alanine aminotransferase; AST, aspartate aminotransferase; CAD, coronary artery disease; eGFR, estimated glomerular filtration rate; FPG, fasting plasma glucose; HDL-C, high-density lipoprotein cholesterol; LDL-C, low-density lipoprotein cholesterol;, MaxAAD, maximum abdominal aortic diameter; SMD, standardized mean difference; TC, total cholesterol; TG, triglycerides; UA, uric acid; UHR, uric acid-to-high-density lipoprotein cholesterol ratio; WBC, white blood cell.

Bold values indicate *P* < 0.05.

Furthermore, generalized additive model (GAM) analysis was used to evaluate the dose-response relationship between UHR and maximum abdominal aortic diameter. A restricted cubic spline (RCS) was used to visually assess the relationship between UHR and the presence of AAA. OR and 95% CI were derived from restricted cubic spline regression, with knots placed at the 5th, 35th, 65th, and 95th percentiles of the UHR distribution. The reference value of UHR was the median of the unmatched group.

Finally, to investigate the consistency of the conclusions, subgroup analyses were performed according to study population (hospital-based vs. community-based), sex (male vs. female), smoking (yes vs. no), hyperuricemia (yes vs. no), hypertension (yes vs. no), CAD (yes vs. no), and diabetes (yes vs. no).

Statistical significance was defined as a two-tailed *P*-value < 0.05. All statistical analyses were carried out using the SPSS, version 24.0 (IBM SPSS 24 Inc), R, version 3.6.1 (The R Project for Statistical Computing, Vienna, Austria), and Empower Stats (X&Y Solutions Inc., Boston, MA, USA).

## Results

### Baseline characteristics

Of the 9,064 study individuals (male 64.3%; mean age, 70.40 ± 7.09 years), the prevalence of AAA was 2.45% (222/9,064).

ROC curve was used to explore the predictive value of UHR with the presence of AAA, and the AUC was 0.66 (95% CI, 0.62–0.69, *P* < 0.001). According to the optimal cut-off value of 17.0% (sensitivity, 61.0%; specificity, 63.0%), the study participants were divided into the following two groups: low-UHR group (UHR < 17.0%, *n* = 5,652) and high-UHR group (UHR ≥ 17.0%, *n* = 3,412).

The baseline characteristics of the two groups are presented in Table 1. The prevalence of AAA was 3.96% in the high-UHR group and 1.54% in the low-UHR group (*P* < 0.001). Significant differences were observed in age, sex, maximum abdominal aortic diameter, smoking status, hypertension, diabetes, CAD, stroke, WBC count, Hb, eGFR, FPG, ALT, AST, UA, TC, TG, LDL-C, and HDL-C levels.

### Association between UHR and presence of AAA

Table 2 presents result for the relationship between UHR and presence of AAA. In the unadjusted model (Model 1), the OR (95% CI) of UHR (as a continuous variable) was 1.04 (1.03–1.05, *P* < 0.001) for the presence of AAA. In the age- and sex-adjusted model (Model 2), a significant positive correlation between UHR and the presence of AAA was found (OR 1.04, 95% CI 1.02–1.05, *P* < 0.001). In the fully adjusted model (Model 3), the positive correlation between UHR and the presence of AAA was still present (OR 1.03, 95% CI 1.01–1.05, *P* < 0.001).

**Table 2:** Association of serum uric acid to high density lipoprotein cholesterol ratio (UHR) on presence of AAA before and after propensity score matching.

Model	Unmatched groups				Propensity score matched groups			
	Continuous UHR	*P*	UHR ≥ 17% vs. <17%	*P*	Continuous UHR	*P*	UHR ≥ 17% vs. <17%	*P*
Model 1^a^ OR (95%CI)	1.04 (1.03–1.05)	<0.001	2.64 (2.01–3.46)	<0.001	1.04 (1.02–1.06)	<0.001	2.01 (1.44–2.80)	<0.001
Model 2^a^ OR (95%CI)	1.04 (1.02–1.05)	<0.001	2.14 (1.62–2.82)	<0.001	1.04 (1.02–1.06)	<0.001	2.06 (1.47–2.87)	<0.001
Model 3^a^ OR (95%CI)	1.03 (1.01–1.05)	<0.001	1.63 (1.18–2.26)	0.003	1.03 (1.01–1.05)	0.008	1.71 (1.19–2.47)	0.004

^a^ Model 1: unadjusted; Model 2: adjusted for age and sex; Model 3: Adjusted for age, sex, smoking, hypertension, diabetes, CAD, stroke, eGFR, WBC, PLT, HB,ALT, AST, FPG, TC, TG, and LDL-C.

Similarly, as a categorical variable, UHR ≥17.0% was associated with a significantly increased risk of AAA in Model 1 (OR 2.64, 95% CI 2.01–3.46, *P* < 0.001), Model 2 (OR 2.14, 95% CI 1.62–2.82, *P* < 0.001), and Model 3 (OR 1.63, 95% CI 1.18–2.26, *P* = 0.003) (Table 2).

Moreover, we categorized the patients into four groups according to the quartiles of UHR to enhance clinical utility: Q1 (UHR < 11.05%), Q2 (11.05% < UHR < 14.92%), Q3 (14.92%≤UHR < 19.86%), and Q4 (UHR ≥ 19.86%). The prevalence of AAA increased from the first to the fourth UHR quartile (1.0% vs. 2.0% vs. 2.4% vs. 4.4%, *P* < 0.001). Compared with the lowest UHR quartile (Q1), the highest UHR quartile (Q4) was independently associated with the presence of AAA in the fully adjusted model (OR 2.13, 95% CI 1.21–3.76,*P* = 0.025) (Supplementary Table 1).

### Propensity score matched analysis

After PSM, 2,452 matched pairs were obtained. The baseline characteristics of the matched groups are shown in Table 1. The prevalence of AAA was 4.32% and 2.20% in the high- and low-UHR groups, respectively (*P* < 0.001). Either as a continuous variable (OR 1.03, 95% CI 1.01–1.05, *P* = 0.008) or as a categorical variable (OR 1.71, 95% CI 1.19–2.47, *P* = 0.004), the association between UHR and the presence of AAA persisted in the fully adjusted model (Model 3) after matching (Table 2).

### The dose-response relationship between UHR and presence of AAA

**Figure 2:**
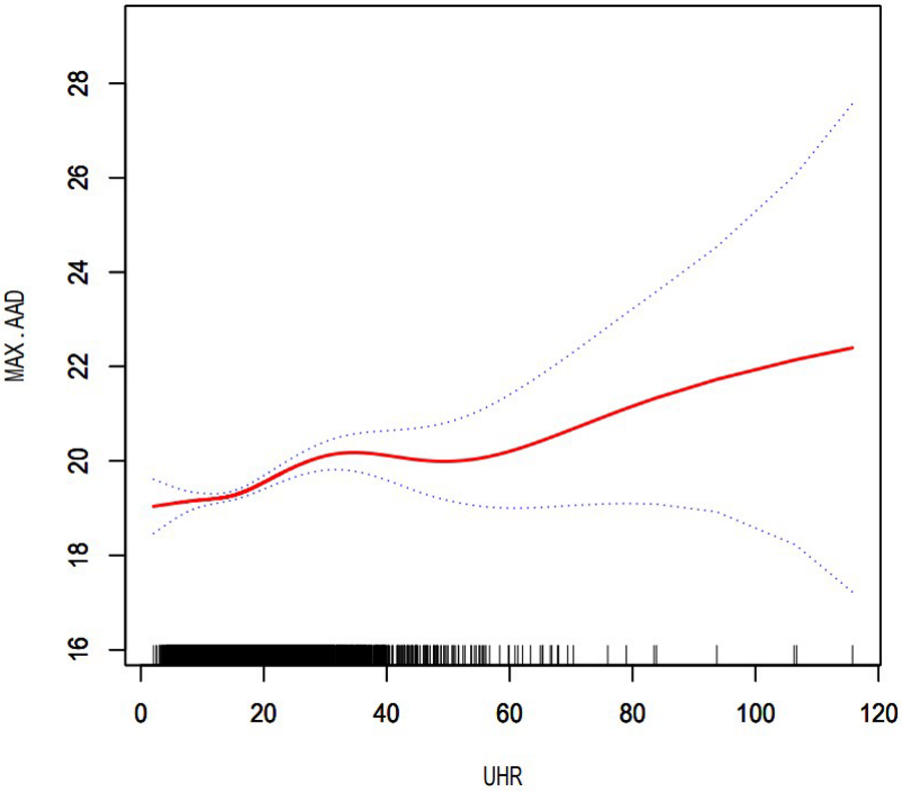
Dose–response relationship between UHR and maximum abdominal aortic diameter. The area between the two dotted lines represents the 95% confidence interval. *P* for non-linearity: <0.001. UHR, uric acid to high-density lipoprotein cholesterol ratio; MAX.AAD, maximum abdominal aortic diameter.

**Figure 3:**
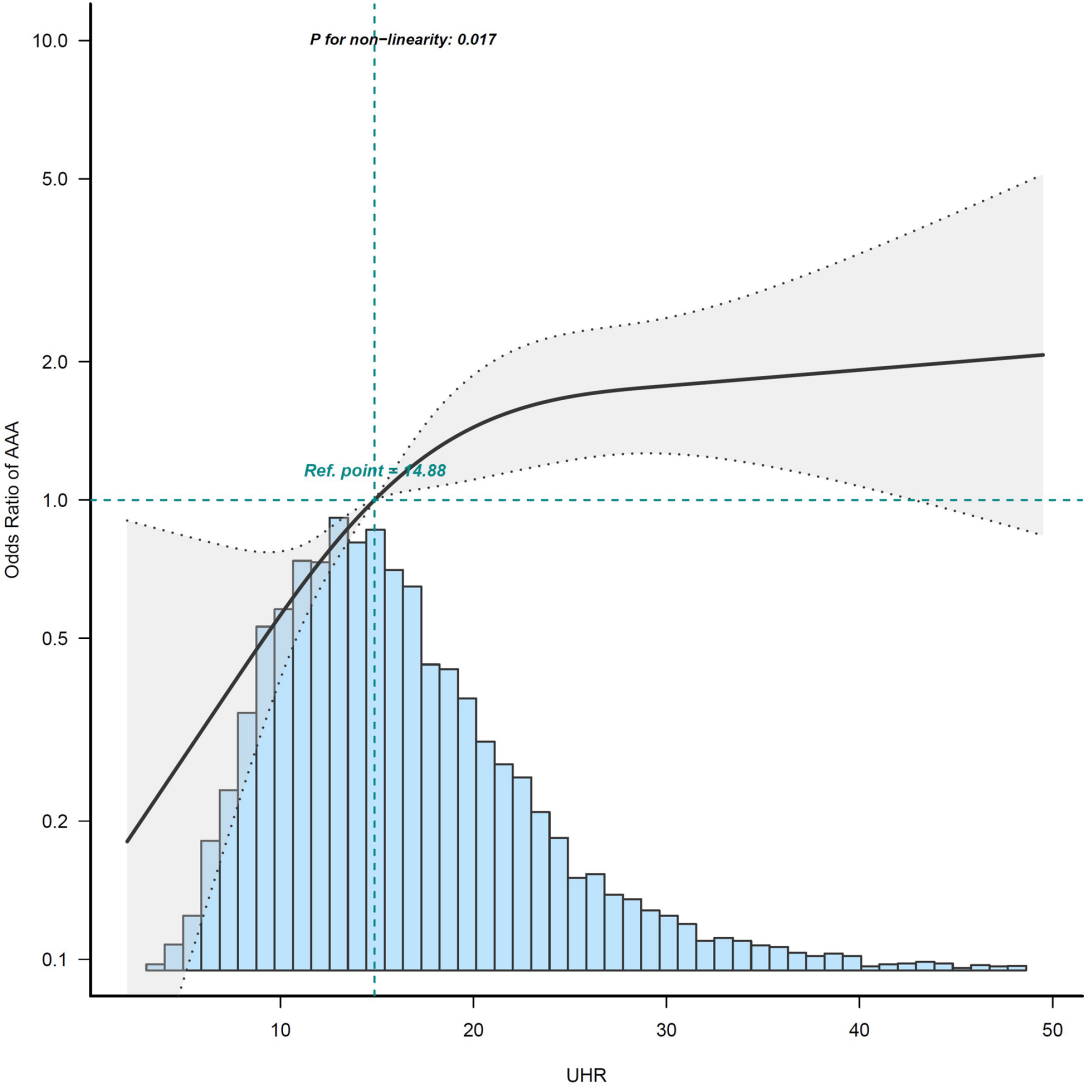
Restricted cubic spline curves for association of UHR on presence of AAA. The area between the two dotted lines represents the 95% confidence intervals (CIs). Odds ratios and 95% CIs were adjusted for the same variables as in model 3. The reference value of the UHR is the median of the reference group. Owing to the small sample and large 95% CI, the highest 0.5% of patients are not shown in the figure. The histogram of the UHR distribution is shown at the bottom of the graph. AAA, abdominal aortic aneurysm; UHR, uric acid to high-density lipoprotein cholesterol ratio.

After controlling for potential confounding factors, as shown in Figure 2, the GAM revealed an S-shaped relationship between the UHR and maximum abdominal aortic diameter (*P* for nonlinearity: < 0.001). The RCS curve showed a nonlinear relationship (*P* for nonlinearity: 0.017) between UHR and the presence of AAA (Figure 3).

### Subgroup analysis

**Figure 4:**
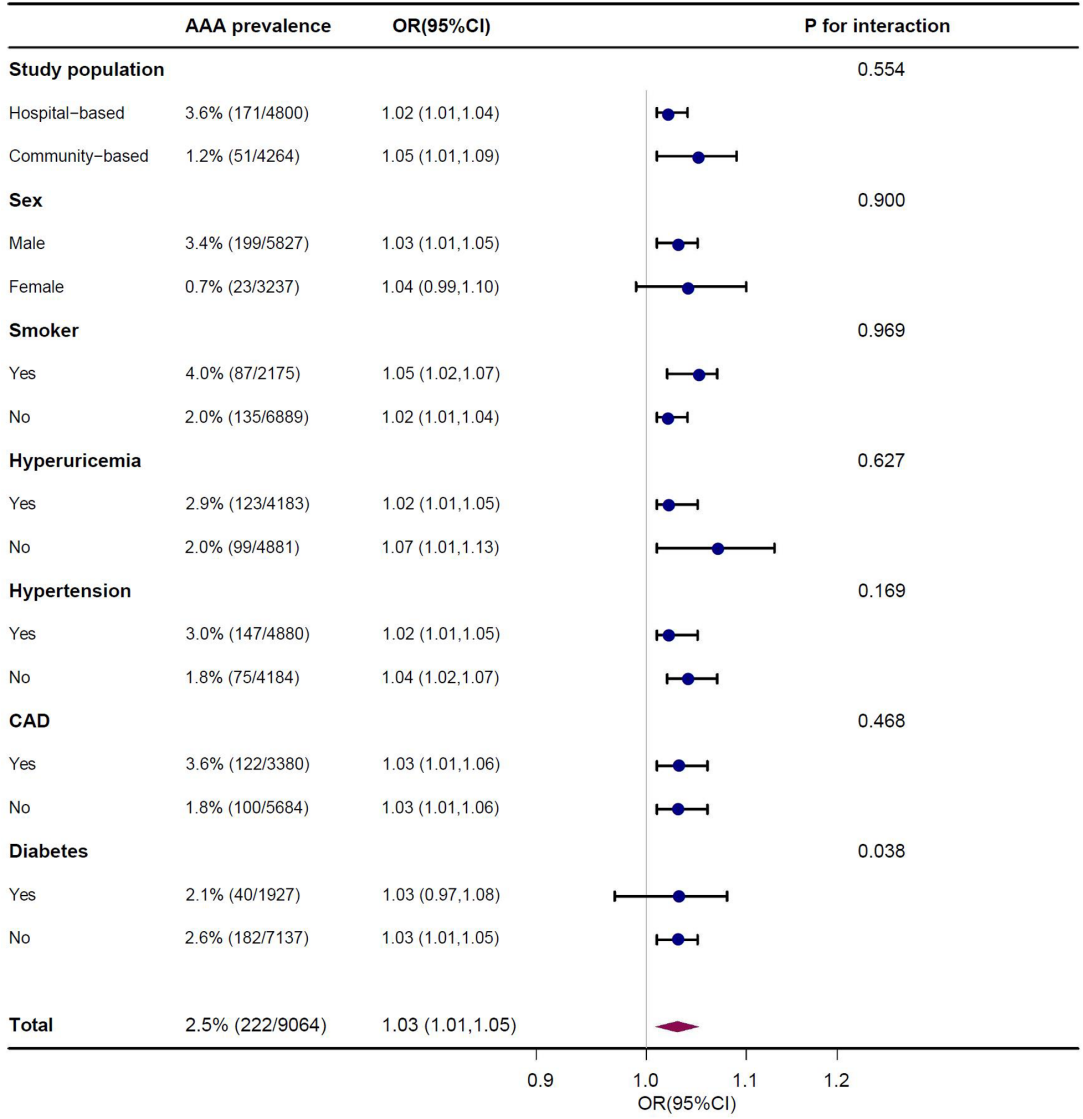
Association of serum uric acid to high density lipoprotein cholesterol ratio (UHR) on presence of AAA in subgroup after multivariable adjustment. Each stratification adjusted for all the factors (age, sex, smoker, hypertension, diabetes, CAD, stroke, eGFR, WBC, PLT, HB, ALT, AST, FPG, TC, TG and LDL-C) except the stratification factor itself.

As shown in Figure 4, subgroup analysis showed that the above covariates (study population, sex, smoking, hyperuricemia, hypertension, and CAD) did not change the relationship between UHR and the presence of AAA (*P* for interaction > 0.05). However, the interaction between UHR and diabetes in relation to the presence of AAA was statistically significant (*P* for interaction = 0.038). The association between UHR and AAA was less significant among individuals with diabetes (OR 1.03, 95% CI 0.97–1.08) compared to those without diabetes (OR 1.03, 95% CI 1.01–1.05).

## Discussion

In the present study, we provide evidence that UHR is positively associated with the presence of AAA, independent of traditional clinical risk factors. After PSM and subgroup analyses, the independent association between UHR and the presence of AAA persisted. More importantly, we observed a nonlinear dose–response relationship between UHR and the presence of AAA. These findings suggest that UHR may be used as a novel and reliable predictor of AAA.

To the best of our knowledge, this is the first study to explore the association between UHR and AAA in both hospital-based and community-based AAA screening programs. It was found that the prevalence of AAA was 2.45% in the present study, which appeared to be lower than that in Caucasian population studies, but not so different from data of Asian populations (10, 12, 13, 25). As the cost-effectiveness of AAA screening is affected by disease prevalence, future screening strategies for AAA should be targeted at high-risk individuals. In addition to traditional clinical risk factors for AAA, such as older age, male sex, smoking history, family history of aneurysms, and so on (26, 27), our results provide evidence for the first time that UHR might be a novel predictor for the presence of AAA in the Chinese population. Compared to the low-UHR group, the prevalence of AAA in the high-UHR group increased significantly (1.54% vs. 3.96%), indicating that screening for AAA could be considered in these high-risk individuals. Replication of the PSM results and subgroup analyses adds to the confidence that the results are reliable.

UA plays an important role in the development and progression of atherosclerosis and stimulates downstream inflammation by stimulating the production of inflammatory factors and intercellular adhesion molecules (28, 29). Although it is well known that inflammation plays an important role in the occurrence and development of AAA (30), few studies have investigated the association between UA and AAA. A previous study found that elevated UA levels were associated with an increased risk of vascular diseases, such as AAA, in patients without metabolic syndrome (31). The relationship between UA and vascular disease may be explained by inflammation, oxidative stress, platelet adhesiveness, and endothelial dysfunction, which are important for the pathogenesis of AAA (32, 33). Moreover, elevated UA levels may elevate blood pressure through their effects on the renal interstitium (34). Experimental and clinical studies have found that UA is also involved in the development of vascular disease through other pathways, including metabolic dysregulation, activation of enzyme channels, and increased insulin resistance (35, 36).

HDL-C affects the cardiovascular system through multiple mechanisms, including anti-inflammatory and antioxidant effects, reversing cholesterol transport to reduce the atherosclerosis burden, and increasing insulin sensitivity (37, 38). A previous study indicated that higher HDL-C levels correlated with decreased AAA growth rates in a screening population (39). Furthermore, a systematic review demonstrated that increased levels of TC and apolipoprotein B have been associated with increased growth rates of AAA (40). A further meta-analysis showed a consistent inverse association of HDL-C with AAA risk (41, 42). Moreover, a recent meta-analysis used Mendelian randomization to provide robust evidence that LDL-C, HDL-C, and TG are likely to play important roles in the etiology of AAA (43).

Additionally, we found a significant interaction between UHR and diabetes, in relation to the presence of AAA. The association between UHR and AAA was less significant among individuals with diabetes than among those without. UHR has recently attracted attention as a novel biomarker for the evaluation of inflammatory and anti-inflammatory interactions (14, 15). These mechanisms may be less important among individuals with diabetes because high glucose levels can lead to similar changes, such as activation of the renin–angiotensin system, increased oxidative stress, and inflammation (44). UHR has also been reported as an independent indicator of metabolic syndrome and diabetes control in patients with type 2 diabetes mellitus (14, 45). Therefore, it is not surprising that the association between UHR and AAA is blunted in individuals with diabetes.

An emerging study found that the ASCVD mortality rate increased as the UHR increased, suggesting that UA is associated with the effect of HDL-C on the prognosis of ASCVD (15). In addition, a cross-sectional study showed that an elevated UA level was strongly associated with HDL-C dysfunction and was found to be a marker for the pre-inflammatory state (14). In our previous study, we found that UA and HDL-C play opposing roles in the atherosclerosis process, and hyperuricemia marks a pre-inflammatory state and impacts the association between HDL-C and carotid atherosclerosis (16). In the present study, we provided evidence that UHR was positively associated with the presence of AAA and that there was a nonlinear relationship between them. In addition, GAM revealed an S-shape upward trend in the dose-response relationship between UHR and maximum abdominal aortic diameter, which demonstrated that UHR was positively associated with dilation of the abdominal aorta. This result is expected to provide a reference for clinicians to control the UHR level. From a therapeutic standpoint, further research is needed in the future to demonstrate reducing the UHR level can significantly reduce the risk of progression to AAA. Our results support the hypothesis that UHR reflects the relative status of serum uric acid and lipids, as well as the extent of damage to the arterial endothelium and inflammation, which in turn can explain the rationality of UHR as a predictor of the occurrence and development of AAA through the above pathophysiological mechanism.

Our study had several limitations. First, it was cross-sectional and could not determine the causal relationship between UHR and AAA. Second, the present study population was composed of Chinese people, which means that the results may not be fully applicable to other ethnic groups. Third, the present study did not have any documentation with respect to dietary patterns in the study population, which could serve as a confounding factor for UHR as they could affect both UA and HDL-C levels.

In conclusion, UHR is positively associated with the presence of AAA, and there is a nonlinear dose-response relationship between them. Thus, UHR may serve as a novel and reliable predictor of AAA. Screening for AAA should be considered in high-risk individuals, especially those with a higher level of UHR.

## Data Availability

The raw data supporting the conclusions of this article will be made available by the authors, without undue reservation.
