# Supplementary material for: The serum uric acid/high-density lipoprotein cholesterol ratio: a novel predictor for the presence of abdominal aortic aneurysm
Source: Front Cardiovasc Med. 2024 Nov 11;11:1481872. doi: 10.3389/fcvm.2024.1481872 (PMC11586378; doi:10.3389/fcvm.2024.1481872)
Supplement: Supplementary file 1 [file Table1.docx]

**Supplementary Table 1. Association of uric acid to high density lipoprotein cholesterol ratio(UHR) by quartiles on presence of AAA.**

| **Model** | **Uric acid to HDL-C ratio quartiles** | | | | |
| --- | --- | --- | --- | --- | --- |
|  | **Q1**  **(UHR＜11.05%)** | **Q2**  **(11.05%≤UHR＜14.92%)** | **Q3**  **(14.92%≤UHR＜19.86%)** | **Q4**  **(UHR≥19.86%)** | **P for trend** |
| **Model 1***  OR (95%CI) | Reference | 2.07 (1.24-3.45) | 2.53 (1.54-4.18) | 4.71 (2.96-7.50) | ＜0.001 |
| **Model 2***  OR (95%CI) | Reference | 1.64 (0.98-2.74) | 1.82 (1.10-3.01) | 3.24 (2.02-5.20) | ＜0.001 |
| **Model 3***  OR (95%CI) | Reference | 1.37 (0.80-2.35) | 1.40 (0.80-2.42) | 2.13 (1.21-3.76) | 0.025 |

Notes: *Model 1: Unadjusted; Model 2: Adjusted for age and sex; Model 3: Adjusted for age, sex, smoking, hypertension, diabetes, CAD, stroke, eGFR, WBC, PLT, HB,ALT, AST, FPG, TC, TG, and LDL-C.
